# Mesenchymal stem cells abrogate experimental asthma by altering dendritic cell function

**DOI:** 10.3892/mmr.2015.3706

**Published:** 2015-04-30

**Authors:** SHAO-LIN ZENG, LI-HUI WANG, PING LI, WEI WANG, JIONG YANG

**Affiliations:** Department of Respiratory Medicine, Zhongnan Hospital, Wuhan University, Wuhan, Hubei 430071, P.R. China

**Keywords:** asthma, mesenchymal stem cells, dendritic cells, chemokine (C-C motif) ligand 17, chemokine (C-C motif) ligand 22, T helper type 2

## Abstract

Mesenchymal stem cells (MSCs) have been investigated in the treatment of numerous autoimmune diseases. However, the immune properties of MSCs on the development of asthma have remained to be fully elucidated. Airway dendritic cells (DCs) have an important role in the pathogenesis of allergic asthma, and disrupting their function may be a novel therapeutic approach. The present study used a mouse model of asthma to demonstrate that transplantation of MSCs suppressed features of asthma by targeting the function of lung myeloid DCs. MSCs suppressed the maturation and migration of lung DCs to the mediastinal lymph nodes, and thereby reducing the allergen-specific T helper type 2 (Th2) response in the nodes. In addition, MSC-treated DCs were less potent in activating naive and effector Th2 cells and the capacity of producing chemokine (C-C motif) ligand 17 (CCL17) and CCL22, which are chemokines attracting Th2 cells, to the airways was reduced. These results supported that MSCs may be used as a potential treatment for asthma.

## Introduction

Mesenchymal stem cells (MSCs) are multipotent cells which abundantly exist in adult bone marrow and adipose tissue (1). MSCs are able to differentiate into numerous lineages of cell types (2); therefore, they are widely used in various fields, including rheumatology, orthopedic surgery, gastroenterology, and transplant surgery (3). Beneficial effects of MSCs have also been demonstrated in a variety of inflammatory disorders, including systemic lupus erythematosis (4), rheumatoid arthritis (5), inflammatory bowel disease (6) and graft-versus-host disease (7). Recently, the role of MSCs in ameliorating the characteristics of asthma has also been reported (8–10); however, the exact mechanism of the inhibitory role of MSCs in asthma remains to be elucidated.

Allergic asthma is a T helper type 2 (Th2) lymphocyte-associated inflammatory airway disease characterized by airway eosinophilia, goblet-cell hyperplasia, variable airway obstruction and bronchial hyper-responsiveness (BHR) to non-specific stimuli. A variety of cells are recruited into the allergically inflamed lungs, including eosinophils, mast cells, T lymphocytes and antigen-presenting dendritic cells (DCs) (11). DCs are the most important antigen-presenting cells (APC) in the immune system, mainly characterized by their capacity to induce primary immune responses (12). In rats, allergen-primed lung DCs induced allergen-specific Th2-mediated immunoglobulin (Ig)G1 with little Th1-directed allergen-specific IgG2b generation (13). Lung DCs are potent regulators of Th2-biased responses to inhalant allergens (14). Pulmonary DCs reside near the epithelium in an immature state, where they sample the airway lumen and are specialized in antigen (Ag) uptake. Upon the triggering of danger signals, DCs upregulate co-stimulatory molecules, such as CD86, and migrate to the mediastinal lymph nodes (MLNs) (15). At arrival in the MLNs, DCs present Ags to naive T cells and induce a polarized T-cell response (15). It is known that the number and maturation state of lung DCs is elevated during secondary immune challenge with allergens and during chronic airway inflammation (16–18). These studies show that maturation, migration to draining lymph nodes and expression of co-stimulatory molecules on DCs are all essential for the priming of T cells and for the development of allergic airway inflammation. A number of studies have focused on the influence of MSCs on DC function (19,20), and demonstrated that MSCs disrupt the three major functions that characterize the transition of DCs from an immature state to a mature state, namely the upregulation of antigen presentation and co-stimulatory molecule expression, the ability to present defined antigens and the capacity of migration (21).

Therefore, the present study aimed to investigate whether transplantation of MSCs was able to suppress experimental asthma, particularly focusing on how MSCs exert effects on DC function.

## Materials and methods

### Experimental animals

Female BALB/c mice and ovalbumin (OVA)-T-cell receptor (TCR) transgenic mice (DO11.10 BALB/c) (n= 6/group), weighing 16–24 g, aged 6–8 weeks, were obtained from the Laboratory Animal Center of Wuhan University (Wuhan, China). The mice were housed in specific pathogen-free conditions, under a 12:12 h light/dark cycle, with *ad libitum* access to water and a standard laboratory diet. The mice were maintained at 18–29°C, with 40–70% relative humidity. Animal care and handling protocols were in accordance with the Guide for the Care and Use of Laboratory Animals (22). All experimental procedures were approved by the Animal Care Committee of Wuhan University (Wuhan, China).

### Isolation and culture of bone marrow-derived MSCs

The mice were sacrificed by cervical dislocation and the femur and tibia were harvested and cleaned of all connective tissue. The bones were then placed in ice-cold isolation medium, which consisted of RPMI-1640 (Invitrogen Life Technologies, Carlsbad, CA, USA), supplemented with 10% heat-inactivated fetal bovine serum (Gibco Life Technologies, Carlsbad, CA, USA), 10% (v/v) equine serum (HyClone, GE Healthcare, Logan, UT, USA), 1% (v/v) penicillin/streptomycin (Gibco Life Technologies), and 1% (v/v) L-glutamine (Gibco Life Technologies). The ends of the bones were then cut to expose the marrow. The cells were flushed out with isolation medium, using a 5 ml syringe with a 27-gauge needle (Dakewei Biotechnology Co., Ltd., Shanghai, China). Cell clumps were disaggregated using a 21-gauge needle and syringe, followed by filtration through a 70 *μ*m nylon mesh filter (Dakewei Biotechnology Co., Ltd). The cells were then centrifuged at 600 × g for 5 min, resuspended in 15 ml isolation medium, and cultured at 37°C and 5% CO_2_. After 24 h, non-adherent cells were removed by washing with sterile PBS, and the isolation medium was replaced. This process was repeated every 3–4 days for 28 days. After 28 days, the cells were removed from the flask by mild trypsinization. Passage 2 cells were seeded at low density (50 cells/cm^2^) and cultured in expansion medium [isolation medium with RPMI-1640 replaced with α-minimum essential medium (Gibco Life Technologies)]. Cells retained their differentiation capacity (23).

### Determination of MSC surface antigen expression

A total of 1×10^5^ cells were incubated with 1:200 dilutions of fluorochrome-conjugated specific or isotype control antibodies for 30 min at 4°C. Specific or isotype control antibodies included: PE/Cy5-rat IgG2b, κ isotype control antibody (Abcam, Cambridge, UK; cat.no. ab18538); PE-rat IgG2a, κ isotype control antibody (BD Pharmingen; cat. no. 554689); PE-armenian hamster IgG2, κ isotype control antibody (BD Pharmingen; cat. no. 550085); APC-armenian hamster IgG1, λ2 isotype control antibody (BioLegend, San Diego, CA, USA; cat. no. 400912); FITC-Rat IgG2a, κ isotype control antibody (Abcam; cat. no. ab18446); biotin-rat IgG2a, κ isotype control antibody (Abcam; cat. no. ab18445). The following antibodies were used: Biotin-conjugated anti-major histocompatibility complex (MHC)I (cat. no. ab25240), fluorescein isothiocyanate (FITC)-conjugated anti-Sca-1 (cat. no. ab25031), phycoerythrin (PE)/Cy5-conjugated anti-CD44 (cat. no. ab25579), FITC-conjugated anti-CD106 (cat. no. ab24853), FITC-conjugated anti-CD45 (cat. no. ab24917), PE-conjugated anti-CD34 (cat. no. ab23830), FITC-conjugated anti-CD117 (cat. no. ab24870), and PE/Cy5-conjugated anti-CD11b (cat. no. ab25533) (Abcam); and FITC-conjugated anti-MHCII (cat. no. 562009) and allophycocyanin (APC)-conjugated anti-CD11c (cat. no. 561119) (BD Pharmingen, San Diego, CA, USA). Fluorescence histograms were obtained by recording 2×10^4^ cells/sample, at a flow rate of ~200 cells/event(s). Experiments were conducted using a FACSCalibur flow cytometer, with CellQuest version 3.3 (BD Biosciences, San Jose, CA, USA), and FlowJo version 6.4.7 (FlowJo, LLC, Ashton, OR, USA).

All MSCs were MHCI^+^, Sca-1^+^, CD44^low^, CD106^low^, MHCII^−^, CD11b^−^, CD11c^−^, CD34^−^, CD45^−^ and CD117^−^. MSCs were used between passages 3 and 10 and rigorous purification and quality control were performed to ensure MSC purity as previously described (24).

### OVA sensitization and airway challenge

BALB/c mice (n=6–8 per group) were immunized on days 0 and 7 by intraperitoneal (i.p.) injection of OVA/alum (10 *μ*g OVA emulsified in 1 mg aluminium hydroxide; grade V; Sigma-Aldrich, St Louis, MO, USA) and were exposed to OVA aerosol challenges (grade III) on days 17–19; aerosol challenges were generated from a jet nebulizer delivering 1% OVA in phosphate-buffered saline (PBS) for 30 min. To examine the effect of MSC transplantation, 24 h prior to the first OVA aerosol challenge, a number of the mice received an intravenous injection of 10^6^ MSCs. The positive control mice received PBS instead of MSCs. The negative control mice were neither sensitized with OVA nor given MSC treatment prior to OVA aerosol challenges. Twenty-four hours after the last OVA exposure, bronchoalveolar lavage fluid (BALF) was collected with 1-ml washes of Ca^2+^- and Mg^2+^-free Hank’s balanced salt solution (HBSS; Invitrogen Life Technologies) supplemented with 0.1 mM EDTA, and slides were prepared with Cytospin III (Shandon Scientific, Pittsburgh, PA, USA) and stained with May-Giemsa (Sigma-Aldrich) for determination of cell counts. Immediately after the collection of BALF, lungs were resected and stored in optimum cutting temperature freezing medium. In certain experiments, MLNs and lungs were digested with collage-nase/DNAse (Sigma-Aldrich) as previously described (25).

### Mouse model of asthma induced by endogenous myeloid airway DCs

Mice received 3 i.p. injections of the plasmacytoid (p)DC-depleting antibody (Ab) Gr-1 (RB6-8C5) to deplete tolerogenic plasmacytoid DCs (pDCs) on days 1, 0, and 1 as described previously (26). On day 0, 800 *μ*g OVA (low in lipopolysaccharide; Worthington Biochemicals, Lakewood, NJ, USA) was injected intrathecally (i.t.) with or without venous injection of 10^6^ MSCs. 10 days later, mice were exposed to OVA aerosols for 30 min on 3 consecutive days and sacrificed 24 h after the last dose. Briefly, the mice were anesthetized with Avertin (2% v/v in PBS; Dakewei Biotechnology Co., Ltd.) and were sacrificed by cervical dislocation. BALF, MLNs and lung tissues were obtained from the mice for further experiments.

### Induction of a mouse model of asthma by adoptive transfer of BMDCs

A model in which sensitization to inhaled OVA was induced by i.t. injection of OVA-pulsed bone marrow-derived mDCs has been previously developed (27). Briefly, bone marrow was flushed with RPMI 1640 from femurs and tibiae of BALB/c mice. Cells were washed, counted and plated in bacteriological 100-mm-diameter Petri dishes. The cell-culture medium used was RPMI 1640 supplemented with gentamicin (60 *μ*g/ml; Invitrogen Life Technologies), 2-mercaptoethanol (5×10^−5^ mol/l; Invitrogen Life Technologies) and 5% fetal calf serum (HyClone). At day 0 of culture, the cells were seeded at a concentration of 2×10^6^/dish in medium containing recombinant murine granulocyte macrophage colony-stimulating factor (GM-CSF; 200 IU/ml; Biolegend, San Diego, CA, USA). On days 3, 6 and 8, the medium was refreshed and GM-CSF was added. More than 90% of the cells were myeloid (m) DCs. On day 9 of culture, cells were pulsed overnight with 100 *μ*g/ml OVA (Worthington Biochemicals) (indicated as OVA-DCs). As a control, DCs were incubated with PBS (indicated as PBS-DCs). Prior to their transfer, a number of DCs were co-cultured with MSCs, while they were pulsed with OVA (indicated as MSC-OVA-DCs). After antigen pulsing, non-adherent DCs were collected, washed 3 times with PBS to remove free OVA and re-suspended in PBS at a concentration of 12.5×10^6^ cells/ml. For *in vivo* experiments, on day 0 under anesthesia, BALB/c mice were instilled through the trachea with 1×10^6^ PBS-treated DCs (PBS-DCs), OVA-pulsed DCs (OVA-DCs) or MSCs-treated OVA-DCs (MSC-OVA-DCs) as described previously (27). Ten days after DC transfer, mice were exposed to a 30-min OVA aerosol once per day for 3 consecutive days and sacrificed 24 h after the last challenge.

### Flow cytometry and cell sorting

For determination of the DC number in the MLNs, MLN cells were stained for DCs [FITC-labeled anti-MHCII (cat. no. ab93561; Abcam), APC-labeled anti-CD11c). The absolute cell number was calculated by multiplying the total leukocyte number by the percentage of each population of interest. For analysis of DC maturation, bone marrow, lung or MLN cell suspensions were stained with FITC-labeled anti-I-Ad/I-Ed; phycoerythrin PE-labeled anti-CD40 (cat. no. 553791), anti-CD80 (cat. no. 553769) and anti-CD86 (cat. no. 553692); and APC-labeled anti-CD11c (cat. no. 561119) Abs (BD Pharmingen). To address migration of lung DCs (25), 80 *μ*l FITC-OVA (10 mg/ml) was administered i.t., with or without venous injection of 10^6^ MSCs. Control mice received 80 *μ*l PBS. After 24 h, migrating DCs were counted in the MLNs as CD11c^+^MHCII^+^ cells carrying FITC^+^ material. In all experiments, dead cells and debris were excluded using propidium iodide (Abcam). Analysis was performed on a FacsCalibur flow cytometer using CellQuest version 3.3 and FlowJo version 6.4.7 software.

### Evaluation of BHR

Assessment of BHR was undertaken 24 h after the last OVA inhalation using a single-chamber, barometric whole-body plethysmograph (Buxco Electronics, Troy, NY, USA) as previously described (28). Briefly, conscious mice were placed in the main chamber and baseline readings were taken and averaged for 3 min. Aerosolized normal saline or acetyl-β-methycholine chloride (MCh; Sigma-Aldrich) at increasing concentrations (1.5625, 3.125, 6.25, 12.5, and 25 mg/ml) were nebulized through an inlet of the main chamber for 3 min. Recordings were taken and averaged for 3 min after each nebulization. The average Heuristic parameter (PenH) values were expressed for each methacholine concentration as the percentage increase over baseline PenH values.

### Activation of OVA-specific naive and effector T cells by mDCs

PBS-treated DCs (PBS-DCs), OVA-pulsed DCs (OVA-DCs) or MSC-treated OVA-DCs (MSC-OVA-DCs) were collected and co-cultured with OVA-specific naive CD4^+^ T cells, which were purified from DO11.10 TCR transgenic mice. In the co-culture system, the ratio of DCs to CD4^+^ T cells from DO11.10 TCR transgenic mice r was 1×10^4^:1×10^5^. In separate experiments, naive DO11.10 T cells were first differentiated for seven days into effector Th2 cells in the presence of interleukin (IL)-4, anti-interferon (IFN)-γ and anti-IL-12 as previously described (29). After washing, these effector Th2 cells were stimulated with DCs as for naive T cells. After 4 days, supernatants were harvested and the concentration of IFN-γ, IL-4, IL-5 and IL-13 cytokines were assayed by specific mouse ELISA kits (Abcam). In separate wells, proliferation was measured by pulsing with [^3^H]thymidine (1 *μ*Ci/well; GE Healthcare Life Sciences, Little Chalfont, UK) for the last 16 h of culture. Cells were then harvested onto glass filters by an automated multi-sample harvester and counted with a dry scintillation counter (Perkin-Elmer, Waltham, MA, USA) and results are presented in cpm (mean ± standard error of the mean) of triplicate cultures.

### Analysis of thymus and activation regulated chemokine (TARC)/chemokine (C-C motif) ligand 17 (CCL17) and macrophage-derived chemokine (MDC)/CCL22 produced by DCs

At day 9 of culture, DCs were collected and seeded at 6×10^5^ cells/ml in 24-well cultures (Nunc, Rorsklide, Denmark) and either not stimulated or pulsed overnight with 100 *μ*g/ml OVA in the presence or absence of MSCs (2×10^5^/ml). Following 24 h, culture supernatants were centrifuged and collected for analysis of TARC/CCL17 and MDC/CCL22 by specific mouse ELISA kits (Abcam).

### Statistical analysis

Values are expressed as the mean ± standard error of the mean. All experimental data were analyzed by SPSS 18.0 (International Business Machines, Armonk, NY, USA). Statistical analysis was performed using one-way analysis of variance followed by Dunnett’s post hoc test. P<0.05 was considered to indicate a statistically significant difference between values.

## Results

### MSC transplantation reduces the cardinal features of asthma

**Figure 1:**
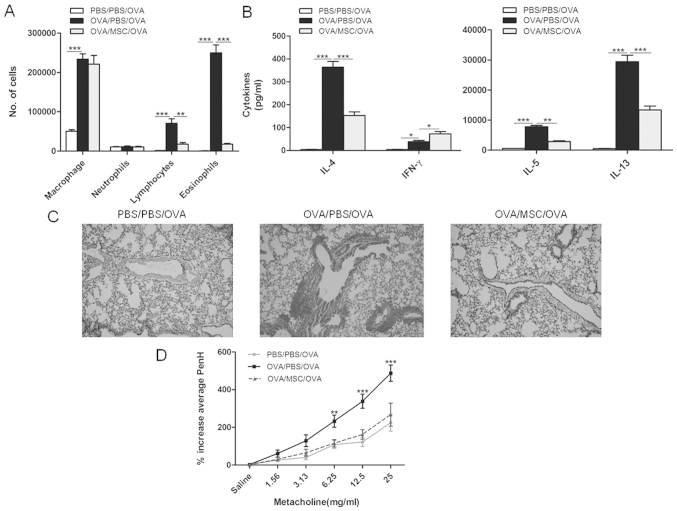
Transplantation of MSCs abrogates the development of asthma. An asthma model was established by intraperitoneal injection of OVA/alum on days 0 and 7 for sensitization. Mice were then exposed to OVA aerosols on days 19–21. Prior to the first aerosol challenge, mice received an intravenous injection of MSCs or control PBS. Labels indicate sensitization/treatment/challenge. (A) Differentiation cell counts in bronchoalveolar lavage fluid were analyzed. (B) Cytokine production in mediastinal lymph node cells re-stimulated *in vitro* for 4 days with OVA. (C) Lung tissue sections stained with hematoxylin and eosin (magnification, ×100). (D) MSCs attenuated airway responsiveness induced by methacholine. Labels indicate immunization/treatment/challenge; n=8 mice per group. Values are expressed as the mean ± standard error of the mean; n=9 mice per group. ^*^P<0.05; ^**^P<0.01; ^***^P<0.001, vs. the OVA/MSC/OVA group. OVA, ovalbumin; PBS, phosphate-buffered saline; MSC, mesenchymal stem cell; IL, interleukin; IFN, interferon; PenH, Heuristic parameter.

Asthma is a chronic pulmonary inflammatory disease characterized by airway and tissue infiltration of eosinophils and other cell types (11). To evaluate the immunoregulatory role of MSCs on pulmonary inflammation, a murine model was generated in which mice were sensitized using i.p. injection of OVA in an alum solution as a Th2 adjuvant. 10 days later, these mice were challenged by exposure to OVA aerosols three times on three consecutive days. After the last challenge, BALF was collected and the various cell types were counted. As expected, upon OVA aerosol challenge, OVA-sensitized mice showed a marked elevation of macrophages, lymphocytes and eosinophils in BALF compared with those in PBS-sensitized mice; however, in the treatment group, MSC transplantation significantly suppressed eosinophil infiltration (Fig. 1A). Cytokine production was also compared between groups in MLN cell cultures (Fig. 1B). Significantly reduced production of IL-4, IL-5 and IL-13 and a minor increase of IFN-γ were found in the MLN cell cultures in the MSC-treated group compared with that in the OVA-challenged group. Cellular tissue infiltration in lung sections in different groups of mice was also assessed. As shown in Fig. 1B, OVA challenge induced marked perivascular and peribronchial inflammatory cell infiltration, and MSC transplantation significantly reduced OVA-induced eosinophilrich leucocyte infiltration and mucus hypersecretion into the airways. Increased airway responsiveness and sensitivity to non-specific stimulation is a major pathological characteristic of asthma. As shown in Fig. 1C, exposure of OVA-sensitized mice to aerosolized allergen resulted in BHR to inhaled MCh compared with that of sham-sensitized mice, but administration of MSCs strikingly attenuated the MCh-induced airflow obstruction. These results demonstrated that MSC transplantation reduced the cadinal features of asthma, including pulmonary inflammation, airway remodelling and bronchial hyperresponsiveness.

### MSC transplantation reduces the presence of DCs in MLN and suppresses DC maturation

**Figure 2:**
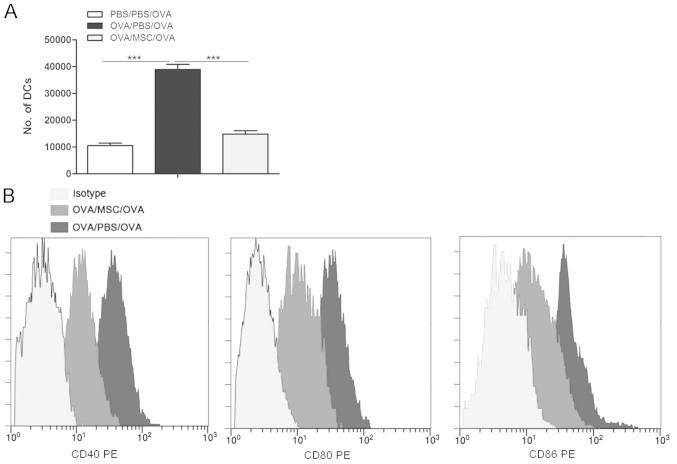
Effect of MSC treatment on the distribution of DCs. (A) The number of DCs in mediastinal lymph nodes was determined by flow cytometry (same experimental conditions as in Fig. 1). Labels indicate sensitization/treatment/challenge. Values are expressed as the mean ± standard error of the mean and were calculated from absolute numbers of cells. ^***^P<0.001. (B) MSCs inhibited the maturation of lung DCs *in vivo*. A single-cell suspension was prepared from the lung tissue, and CD11c^+^MHCII^hi^ lung DCs were analyzed for their expression of CD40, CD80 and CD86. Representative results from one out of four experiments are shown. PE, phycoerythrin; DC, dendritic cell; hi, high; OVA, ovalbumin; PBS, phosphate-buffered saline; MSC, mesenchymal stem cell; MHC, major histocompatibility complex.

As administration of MSCs prior to allergen challenge abolished the characteristics of asthma, it was hypothesized that this response may result from direct alteration of DC function. The total number of DCs (MHCII^high^CD11c^high^) in MLNs was determined 24 h after the last OVA challenge. As shown in Fig. 2A, in OVA-sensitized mice, OVA challenge led to an increase of DCs in the MLNs compared with those in sham-sensitized mice. Of note, intravenous injection of MSCs prior to OVA challenge markedly reduced this increase (Fig. 2A).

The maturation status of DCs is associated with their capacity to initiate immune responses. Next, it was investigated whether MSC transplantation affected lung DC maturation. As shown in Fig. 2B, transplantation of MSC markedly reduced the expression of the co-stimulatory molecules CD40, CD80 and CD86 on CD11c^+^MHCII^+^ lung DCs from allergen-challenged mice.

### MSC transplantation reduces lung DC migration to the MLNs

**Figure 3:**
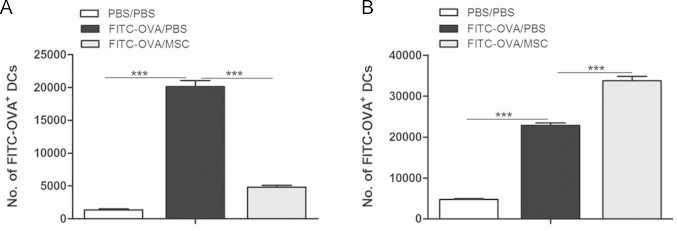
MSCs suppress lung DC migration to the MLNs. (A) On day 0, naive mice were instilled intrathecally with FITC-OVA with or without tail intravenous injection of MSCs. On day 1, the presence of FITC^+^ migrating DCs in MLNs was analyzed by flow cytometry. (B) In the same mice, lung DCs were also counted. Lungs were enzymatically digested and stained for the presence of FITC^+^MHCII^+^CD11c^+^ DCs. Values are expressed as thee mean ± standard error of the mean (n=8). ^***^P<0.001. FITC, fluorescein isothiocyanate; DC, dendritic cell; OVA, ovalbumin; PBS, phosphate-buffered saline; MSC, mesenchymal stem cell; MHC, major histocompatibility complex; MLN, mediastinal lymph node.

The present study examined whether MSC transplantation was able to reduce the migratory ability of DCs. Mice received i.t. injection of OVA-FITC 24 h after instillation, and FITC^+^ DCs (CD11c^+^ and MHC-II^+^ cells) in the MLNs were counted. As shown in Fig. 3A, MSC transplantation significantly inhibited the migration of lung DCs to the MLNs, and significantly more FITC^+^ DCs were retained in the lungs after MSC treatment (Fig. 3B).

### Effect of MSCs on Th2 priming induced by lung mDCs

**Figure 4:**
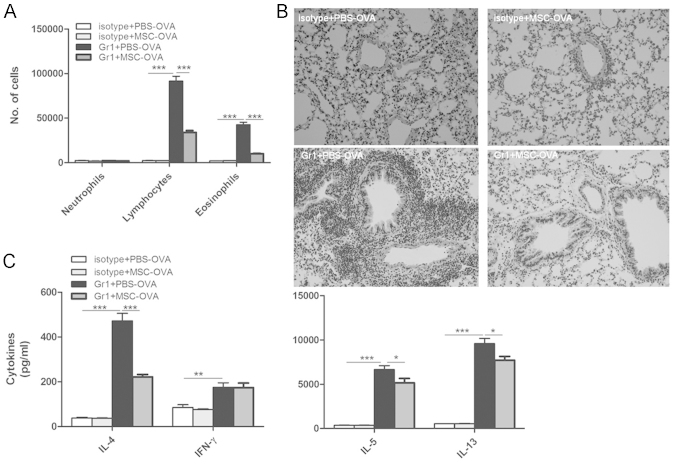
Transplantation of MSCs prevents sensitization induced by DCs. On day 0, mice received an intrathecal injection of OVA with or without intravenous injection of MSCs. From days 1 to 2, mice were injected intraperitoneally with anti-Gr-1 antibodies to deplete plasmacytoid DCs or isotype control antibodies. Ten days later, mice were exposed to OVA aerosols three times on three consecutive days. (A) Bronchoalveolar lavage fluid was analyzed by flow cytometry. (B) Hematoxylin and eosin staining of lung sections (magnification, x100). (C) Mediastinal lymph node cells were re-stimulated *in vitro* for 4 days with OVA, and cytokines were measured in the supernatant. Values are expressed as the mean ± standard error of the mean (n=8). ^*^P<0.05; ^**^P<0.01; ^***^P<0.001. OVA, ovalbumin; PBS, phosphate-buffered saline; MSC, mesenchymal stem cell; DC, dendritic cell; IL, interleukin; IFN, interferon.

To elucidate whether MSC administration is able to suppress the function of lung DCs *in vivo*, a model was used in which sensitization to OVA occurs via the airways by endogenous mDCs (30). In the preceding experiments, mice were depleted of pDCs with anti-Gr-1 Abs and then primed with a single injection of 800 mg of LPS-low OVA. In the anti-Gr-1-treated group, but not in the isotype-treated control group, OVA inhalation led to the development of cardinal features of asthma, as shown by eosinophilia in the BALF (Fig. 4A), goblet cell hyperplasia in the lung (Fig. 4B) and Th2 cytokine production in the MLNs (Fig. 4C). However, mice depleted of pDCs and receiving MSC transplantation at the time of OVA priming did not develop any signs of airway inflammation and lymph node cytokine production was reduced in these animals.

### MSC reduces the potential of mDCs to induce Th2 development in vivo

**Figure 5:**
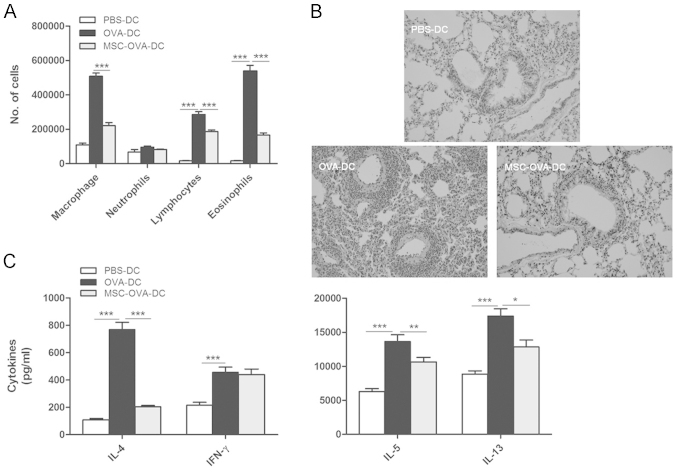
MSC treatment inhibits the potential of DCs to induce asthmatic inflammation. (A and B) On day 0, mice received an intrathecal injection of un-pulsed DCs (PBS-DC), OVA-pulsed DCs (OVA-DC) or MSC-treated OVA-DCs (MSC-OVA-DC). From days 10–13, all mice were exposed to OVA aerosols. (A) Bronchoalveolar lavage fluid was analyzed by flow cytometry. (B) Hematoxylin and eosin staining of lung sections (magnification, ×100). (C) MLN cells were re-stimulated *in vitro* for 4 days with OVA, and cytokines were measured using ELISA. Values are expressed as the mean ± standard error of the mean (n=8). ^*^P<0.05; ^**^P<0.01; ^***^P<0.001. OVA, ovalbumin; PBS, phosphate-buffered saline; MSC, mesenchymal stem cell; DC, dendritic cell; IL, interleukin; IFN, interferon.

MSC transplantation resulted in reduced allergic sensitization and may have resulted from direct or indirect influence on DCs to prime Th2 differentiation *in vivo*. To rule out any indirect effects, mDCs were pre-treated with MSCs prior to their i.t. transfer into naive mice. As previously reported, adoptive i.t. transfer of OVA-pulsed mDCs led to Th2 priming and subsequent development of features of asthma upon OVA aerosol challenge 10 days later, a function associated with the number of DCs injected (27). As expected, mice receiving unpulsed DCs showed a lower amount of inflammatory cells accumulated in the BALF (Fig. 5A) and lung tissue (Fig. 5B) compared with mice that had received pulsed DCs. By contrast, mice that had received OVA-pulsed DCs showed asthmatic inflammation characterized by marked cellular infiltration of lymphocytes and eosinophils in the BALF (Fig. 5A) as well as in the peribronchial and perivascular area (Fig. 5B). Pre-treatment of OVA-pulsed mDCs with MSCs significantly reduced the capacity of these cells to induce eosinophilic airway inflammation and goblet cell hyperplasia in mice (Fig. 5B). This was accompanied by a significant decrease in IL-4, IL-5 and IL-13 levels in the MLNs, while the concentration of IFN-γ was not significantly changed (Fig. 5C).

### Effect of in vitro MSC treatment on the capacity of DCs to activate and polarize Ag-specific T cells in vitro

**Figure 6:**
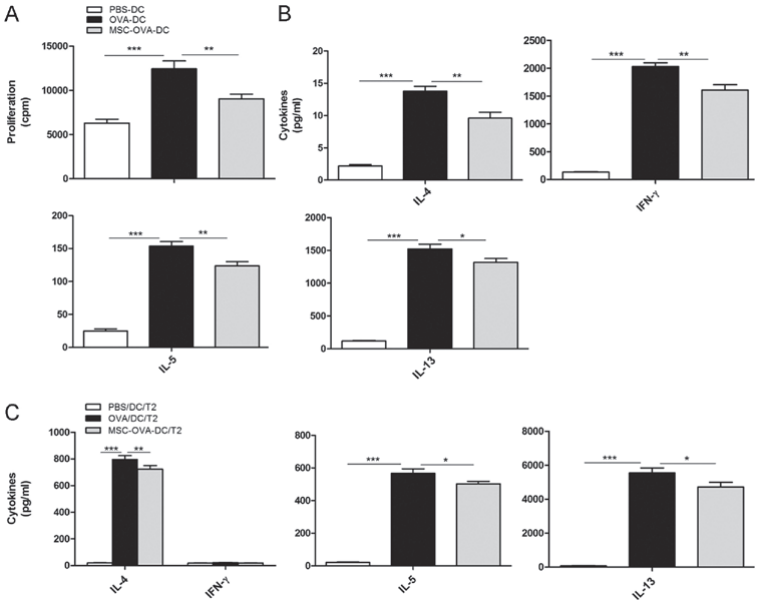
MSCs suppressed DCs to activate and polarize antigen-specific T cells *in vitro*. Un-pulsed DCs (PBS-DC), OVA-pulsed DCs (OVA-DC) or MSC-treated OVA-DC (MSC-OVA-DC) were collected and co-cultured with (A and B) OVA-specific naive CD4^+^ T or (C) *in vitro*-differentiated Th2 cells. (A) Cell proliferation was assessed by [^3^H]thymidine (1 *μ*Ci/well) uptake in an 16-hour pulse. Values are expressed as the mean ± standard error of triplicate cultures (n=8). (B and C) Cytokines were measured in supernatants by ELISA. ^*^P<0.05; ^**^P<0.01; ^***^P<0.001. OVA, ovalbumin; PBS, phosphate-buffered saline; MSC, mesenchymal stem cell; DC, dendritic cell; IL, interleukin; IFN, interferon; T2/Th2, T helper type 2 cells.

As MSC treatment profoundly impaired the migration of DCs to MLNs, it was next investigated whether this could impact the potential of DCs to activate naive T cells. To this point, the effect of MSCs on DC-driven OVA-specific T-cell (DO11.10) proliferation and cytokine production had been tested *in vitro*. As shown in Fig. 6A, MSC-treated OVA-DCs (MSC-OVA-DCs) induced the proliferation of OVA-specific naive T cells to a lesser extent than OVA-pulsed DCs (OVA-DCs). Moreover, T cells stimulated by MSC-OVA-DCs produced lower levels of Th2 cytokines than T cells stimulated with OVA-DCs (Fig. 6B).

In a separate experiment, MSC-OVA-DCs were co-cultured with *in vitro*-differentiated DO11.10 Th2 cells, which were obtained by stimulating MLN cells with OVA in the presence of IL-4, anti-IFN-γ and anti-IL-12 (29). As shown in Fig. 6C, pre-treatment of bone marrow-derived MSCs with MSCs markedly reduced the production of Th2 cytokines by stimulating effector Th2 cells.

### MSCs suppress the production of TARC/CCL17 and MDC/CCL22 by DC

**Figure 7:**
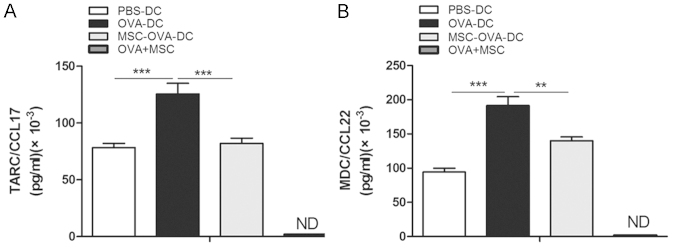
MSCs suppressed the production of (A) TARC/CCL17 and (B) MDC/CCL22 by DCs. Supernatants of control unpulsed DCs (PBS-DC), OVA-pulsed DCs (OVA-DC), DCs pulsed with OVA in the presence of MSC (MSC-OVA-DC) or MSCs pulsed with OVA (OVA+MSC) were assayed for TARC/CCL17 and MDC/CCL22 by ELISA. alues are expressed as the mean ± standard error of triplicate experiments (n=6). ^**^P<0.01; ^***^P<0.001. ND, not detectable; OVA, ovalbumin; PBS, phosphate-buffered saline; MSC, mesenchymal stem cell; DC, dendritic cell; TARC, thymus and activation regulated chemokine; CCL17/22, chemokine (C-C motif) ligand 17/22.

To investigate the effects of MSCs on TARC/CCL17 and MDC/CCL22 production by DCs, supernatants of non-pulsed-DCs (PBS-DCs), DCs pulsed with OVA in the presence or absence of MSCs (OVA-DCs and MSC-OVA-DCs, respectively) and OVA-pulsed MSCs (OVA+MSCs) were collected, and the concentrations of TARC/CCL17 and MDC/CCL22 were determined by ELISA. As shown in Fig. 7, DCs, upon stimulation with OVA, produced large quantities of TARC/CCL17 and MDC/CCL22 compared with un-pulsed DCs; however, this upregulation was signifi-cantly inhibited by MSCs. In addition, it was found that when MSCs were cultured independently and stimulated with OVA, TARC/CCL17 and MDC/CCL22 levels were undetectably low. This indicated that MSCs were able to reduce the TARC/CCL17 and MDC/CCL22 production by DCs, but MSCs could not produce any TARC/CCL17 and MDC/CCL22.

## Discussion

The present study, clearly demonstrated the immune regulatory role of MSCs. To date, the role of MSCs in immune-mediated disorders such as asthma has not been elucidated. The present study clarified that MSCs potently suppressed Ag-induced immune responses through inhibition of DCs.

Given their vast proliferative potential, immunosuppressive properties and the ease of access to available tissue sources, therapies with autologous or allogeneic MSCs have been tested in a variety of immune-mediated disease models, including experimental allergic encephalomyelitis (31), diabetic NOD/scid mice (32), collagen-induced arthritis (33) and several murine models of lupus (34). The role of MSCs in treating asthma has gained significant interest. In a study by Kapoor *et al* (35) MSCs suppressed the proliferation of dust mite (DM)-challenged peripheral blood mononuclear cells (PBMCs) from allergic asthmatic subjects but not from allergic subjects without asthma. Repeated exposure to low-dose DMs and MSCs, as well as pre-conditioning of PBMCs with MSCs, caused refractoriness to DMs. Firinci *et al* (36) reported that MSCs migrated to lung tissue and ameliorated bronchial asthma in a murine model. In an experimental toluene diisocyanate (TDI)-induced animal model of severe asthma, MSC transfer significantly reduced the TDI-induced increase in the inflammatory index as well as numbers of eosinophils and neutrophils in BALF; the MSC transfer also significantly reduced the number of goblet cells, collagen deposition and immune staining for smooth muscle actin and proliferating cell nuclear antigen with concomitant normalization of the airway response to MCh. In the present study, it was demonstrated that transplantation of MSCs during the challenge phase strongly reduced the cardinal features of asthma, including eosinophilic inflammaion, goblet cell hyperplasia, Th2 cytokine production and, most importantly, BHR to inhaled MCh. Studies have reported that intravenously introduced human MSCs localize in the lung prior to dispersing into the peripheral tissues and seemingly homing to injured tissue (37,38). However, how MSCs perform out their ’regulatory’ role in asthma remains to be elucidated.

It has known that pulmonary DCs are crucial mediators in regulating immune responses in the lung and that these DCs bridge the innate and adaptive immune response. In asthma, for example, DCs are important in the induction and maintenance of the disease (15). Depletion of airway DCs during secondary challenge in sensitized mice abolished all cardinal features of asthma (including airway eosinophilia, goblet cell hyperplasia and BHR to MCh), an effect that was completely restored by adoptive transfer of wild-type DCs (29). DCs are crucial as they can locally activate Th2 effector cells in the airway wall by providing chemotactic cues for Th2 cells (CCL17 and CCL22) and by delivering MHC and co-stimulatory signals (39–42). In addition, at times of allergen challenge, DCs migrate from the site of allergic inflammation to MLNs to stimulate new rounds of division in re-circulating central memory cells or from naive T cells, thus feeding the inflammatory response with new waves of effector cells (18,43–45). The present study found that transplantation of MSCs decreased the number of mDCs in MLNs of allergen-challenged mice. Although a reduction in lung inflammation may have been responsible for reduced input of immigrating lung-derived DCs in MLNs, it was also observed that MSC transplantation suppressed the migration of DCs fluorescently labeled with OVA to MLNs in naive mice, concomitant with an accumulation of DCs in the lung compartment. A previous *in vitro* study demonstrated that DCs matured in the presence of MSCs and showed significantly reduced migration to MLNs; furthermore, MSCs prevented loss of expression of the tissue-anchoring protein E-cadherin (21), which was downregulated when DCs migrated to the local lymph node. As the level of maturation is the hallmark of DC activity and is critical for priming naive T-cell responses, the present study assessed the maturation status of pulmonary DCs upon encountering a soluble allergen. The results showed that pulmonary DCs from OVA-sensitized and -challenged mice treated with MSCs showed reduced expression of CD40, CD80 and CD86 compared with those in the PBS-treated asthma group. It was recently shown that CD80/CD86 co-stimulation on DCs is necessary for differentiation of Th2 cells from naive T cells and for re-stimulation of effector Th2 cells in the lung (40,46,47). Thus, in the present study, MSC transplantation hampered DC maturation, leading to the reduced capacity of priming the naive T-cell response with concomitant reduced airway inflammation.

In pulmonary tissue, various DC subsets can be found, based on anatomical location and function (48,49): Classic/myeloid (c)DCs, subdivided into CD11b^+^ and CD11b-cDCs and pDCs. CD11b-cDCs are located adjacent to the epithelium and extend their dendrites between epithelial cells to sample the airway lumen (50), whereas CD11b^+^cDCs are located underneath the epithelium and pick up Ags that have passed the basal membrane. CD11b^−^cDCs were shown to be prolific producers of inflammatory chemokines, and in allergen-challenged mice, CD11b^−^cDCs were shown to produce the highest amounts of Th2 cell-attracting chemokines (51). cDCs, however, are important in inducing sensitization, while pDCs are involved in the induction of immune tolerance; depletion of pDCs during exposure to harmless Ags was previously shown to induce sensitization (26). Inhalation of LPS-low OVA is normally a tolerogenic event, in which pDCs inhibit the potential of mDCs to prime effector Th2 cells (39). In the present study, pDCs were depleted, which abolished this tolerogenic response and led to a robust Th2 priming by mDCs. Transplantation of MSCs reduced the development of the Th2 immune response, and consequently, asthma did not develop upon repeated OVA challenge.

The effects of MSCs on DCs may be direct or indirect. In support of the direct effects of MSCs on DCs, the present study found that *in vitro* pre-treatment of OVA-pulsed mDCs with MSCs prior to transfer to the airways significantly reduced their potential to induce Th2 priming, and consequently, asthmatic inflammation did not develop. The *in vitro* study also indicated that when DCs were pre-treated with MSCs, they were defective in priming naive and memory T cells due to an intrinsic defect in T-cell stimulatory capacity. When OVA-pulsed bone marrow-derived MSCs were co-cultured with OVA-specific T cells from naive mice, reduced proliferation and production of Th1 and Th2 effector cytokines were observed. Furthermore, pre-treatment of DCs with MSCs inhibited their potential to stimulate primed effector Th2 cells generated under polarized Th2 conditions *in vitro*. Similar effects were also reported by English *et al* (21), who used an antigen-specific T-cell proliferation assay and specific peptide:MHCII antigen display to demonstrate that MSCs interfered with the antigen presentation ability of DCs.

Previous studies showed that TARC/CCL17 and MDC/CCL22 induce the selective migration of Th2 cells but not Th1 cells through triggering of CCR4 (52), and the BALF levels of TARC/CCL17 and MDC/CCL22 but not Th1-selective chemokines are increased upon allergen challenge to the lung (53,54). Furthermore, *in vitro* house DM exposure of DCs from house DM-allergic patients but not that of healthy controls caused CCL17 and CCL22 release resulting in chemoattraction of polarized human Th2 cells in a CCR4-dependent manner (55). The present *in vitro* study also demonstrated that when DCs were co-cultured with MSCs, their capacity of producing CCL17 and CCL22 was significantly suppressed. This may explain why in the murine model, transplantation of MSCs suppressed CCL17 and CCL22 production by DCs, leading to reduced recruitment of Th2 cells and the concomitantly reduced level of airway inflammation.

In conclusion, the present study demonstrated that MSCs inhibit Th2-mediated cardinal features of asthma by altering the function of lung DCs, including the state of maturation, capacity of migration, ability of antigen presentation and the capacity of chemokine production. MSCs may be applicable as novel therapeutics for the treatment of airway remodeling and inflammation associated with chronic asthma.
